# On the relationship between metabolic capacities and *in vivo* viscoelastic properties of the liver

**DOI:** 10.3389/fbioe.2022.1042711

**Published:** 2023-01-09

**Authors:** Mehrgan Shahryari, Sarah Keller, David Meierhofer, Iwona Wallach, Yasmine Safraou, Jing Guo, Stephan R. Marticorena Garcia, Jürgen Braun, Marcus R. Makowski, Ingolf Sack, Nikolaus Berndt

**Affiliations:** ^1^ Department of Radiology, Charité—Universitätsmedizin Berlin, Corporate Member of Freie Universität Berlin and Humboldt-Universität zu Berlin, Berlin, Germany; ^2^ Mass Spectrometry Facility, Max Planck Institute for Molecular Genetics, Berlin, Germany; ^3^ Institute of Computer-Assisted Cardiovascular Medicine, Charité—Universitätsmedizin Berlin, Corporate Member of Freie Universität Berlin and Humboldt-Universität zu Berlin, Berlin, Germany; ^4^ Institute of Medical Informatics, Charité—Universitätsmedizin Berlin, Corporate Member of Freie Universität Berlin and Humboldt-Universität zu Berlin, Berlin, Germany; ^5^ Department of Diagnostic and Interventional Radiology, Technical University of Munich, Faculty of Medicine, Munich, Germany

**Keywords:** viscoelasticity, MRE, PET, stiffness, liver metabolism, proteomics, reserve capacity, hepatic function model

## Abstract

The liver is the central metabolic organ. It constantly adapts its metabolic capacity to current physiological requirements. However, the relationship between tissue structure and hepatic function is incompletely understood; this results in a lack of diagnostic markers in medical imaging that can provide information about the liver’s metabolic capacity. Therefore, using normal rabbit livers, we combined magnetic resonance elastography (MRE) with proteomics-based kinetic modeling of central liver metabolism to investigate the potential role of MRE for predicting the liver’s metabolic function *in vivo*. Nineteen New Zealand white rabbits were investigated by multifrequency MRE and positron emission tomography (PET). This yielded maps of shear wave speed (SWS), penetration rate (PR) and standardized uptake value (SUV). Proteomic analysis was performed after the scans. Hepatic metabolic functions were assessed on the basis of the HEPATOKIN1 model in combination with a model of hepatic lipid-droplet metabolism using liquid chromatography–mass spectrometry. Our results showed marked differences between individual livers in both metabolic functions and stiffness properties, though not in SUV. When livers were divided into ‘stiff’ and ‘soft’ subgroups (cutoff SWS = 1.6 m/s), stiff livers showed a lower capacity for triacylglycerol storage, while at the same time showing an increased capacity for gluconeogenesis and cholesterol synthesis. Furthermore, SWS was correlated with gluconeogenesis and PR with urea production and glutamine exchange. In conclusion, our study indicates a close relationship between the viscoelastic properties of the liver and metabolic function. This could be used in future studies to predict non-invasively the functional reserve capacity of the liver in patients.

## 1 Introduction

The liver is the key metabolic organ of the human body. It has a wide range of metabolic functions including homeostatic regulation of numerous plasma metabolites (glucose, amino acids and lipoproteins), detoxification of endogenously formed metabolic end products (e.g., ammonia, urea), and storage of nutrients in the form of glycogen or triacylglycerol. It continuously adapts its metabolic capacities to the current physiological status of the individual. For example, after a carbohydrate-rich meal, the liver takes up a substantial amount of glucose from the plasma; this is then transiently converted into glycogen and triacylglycerol. Conversely, under fasting conditions the liver produces glucose by phosphorolysis of glycogen and synthesis *de novo* from amino acids, lactate and glycerol to prevent a potentially life-threatening drop in plasma glucose below 55 mg/dl (Mathew et al., 2022). Metabolic alterations become permanent as a result of long-term life habits such as overnutrition, as in metabolic syndrome (MetS). MetS–with its hallmarks of obesity, insulin resistance and dyslipidaemia (Bussler et al., 2017)—is an epidemic disease that is often triggered by childhood overnutrition. Non-alcoholic fatty liver disease (NAFLD) is recognized as the hepatic manifestation of MetS (Lonardo et al., 2015) and can progress silently into liver fibrosis and cirrhosis without apparent clinical symptoms (Sivell, 2019). Today, non-alcoholic steatohepatitis is the most common etiology among waiting-list candidates for liver transplantation (Wong and Singal, 2020). Nevertheless, early diagnosis and differentiation of liver diseases remains a major challenge. While cirrhosis and liver tumors represent the end-stage of liver pathology, with life-threatening complications and clear clinical manifestation, most patients with NAFLD have a stable and relatively benign fatty liver for a long time (Caldwell and Argo, 2010).

Evaluation of liver function is a diagnostic challenge because there are only few techniques that can directly quantify metabolic functions *in vivo*. Quantitative liver function test, which use specific metabolites, such as indocyanine green clearance test or methacetin breath-test are complex and costly, and they entail considerable discomfort for the subject (Lalazar et al., 2008; Seyama and Kokudo, 2009). Furthermore, they assess only very specific pathways under very restricted conditions and cannot be used for a general evaluation of metabolic capacities (Vos et al., 2014; De Gasperi et al., 2016; Gorowska-Kowolik et al., 2017). New computational methods such as proteomics-based evaluation of metabolic liver function by large-scale kinetic modelling allows the assessment of metabolic capacities under a wide range of physiological and pathological conditions (Berndt et al., 2018a; Berndt et al., 2019; Berndt et al., 2020; Kespohl et al., 2020; Berndt et al., 2021a; Berndt et al., 2021b). However, these require biopsies, which are limited in follow-up examinations owing to pain, risk of bleeding and sampling errors (Ratziu et al., 2005; Boyd et al., 2020). Despite advances in quantitative medical imaging, there is still no reliable non-invasive imaging marker that can detect metabolic changes occurring in seemingly healthy livers during the ‘silent progression’ phase of NAFLD (Chalasani et al., 2018). Positron emission tomography (PET) is a versatile technique for the assessment of glucose metabolism in lesions or neurodegenerative diseases, where it can be used to investigate changes in various neurotransmitter systems, neuroinflammation, and protein aggregates that characterize the disease. However, PET scans are expensive, they result in exposure of the patient to ionizing radiation and they address only specific metabolic pathways that are not necessarily fully suited for assessment of the liver’s metabolic capacity. Ultrasound-based elastography and magnetic resonance elastography (MRE) have become successful in addressing structural changes such as like degree of fibrosis and steatosis, and they therefore have the potential to replace biopsy-based histological evaluation of structural alteration (Singh et al., 2015; Castera et al., 2019; Hudert et al., 2021; Qu et al., 2021; Selvaraj et al., 2021). Liver fibrosis increases liver stiffness through accumulation and cross-linking of matrix proteins (Huwart et al., 2008; Singh et al., 2015; Reiter et al., 2020). However, beyond fibrosis, the liver’s biomechanical properties are also affected by non-fibrotic alterations such as prandial states (Yin et al., 2011; Jajamovich et al., 2014; Petzold et al., 2019; Obrzut et al., 2021), hydration (Ipek-Ugay et al., 2016; Dittmann et al., 2017), blood perfusion (Ipek-Ugay et al., 2016; Meyer et al., 2022a), cell hypertrophy (Garczyńska et al., 2020), fat accumulation (Hudert et al., 2019) or inflammation (Qu et al., 2021; Selvaraj et al., 2021), making elastography unspecifically sensitive to a variety of pathophysiological processes that occur in the course of NAFLD. Only little is known about the correlation between liver biomechanical parameters and liver metabolism. In cancer patients, liver function has been correlated with stiffness measured by ultrasound-based elastography (Heucke et al., 2019) and with viscoelastic parameters measured by MRE (Lin et al., 2022). However, it remains unclear which specific metabolic function may influence viscoelastic parameters in the liver and whether apparently normal livers display a range of biomechanical properties that are correlated with the variability of metabolic functions.

Therefore, in this study we used a comprehensive kinetic model of central liver metabolism (Berndt et al., 2018a) to characterize metabolic states and capacities of healthy rabbit livers on the basis of proteomic data. We performed *in vivo* MRE in a clinical hybrid PET/MRI scanner to investigate the relationship between viscoelasticity and liver metabolism. In addition, we analyzed the *in vivo* glucose metabolism using ^18^F-fluorodesoxyglucose (FDG)-based PET to test the sensitivity of this imaging marker toward small variations in liver metabolism in correlation with MRE.

## 2 Materials and methods

### 2.1 Animal model

This study and all procedures involving animals were approved by the local authority (Landesamt für Gesundheit und Soziales Berlin, Reg. No. 0178/17). The experimental protocols were performed in accordance with the regulations and guidelines of the Federation of Laboratory Animal Science Association (FELASA) as well as our institutional guidelines.

Nineteen female New Zealand white rabbits (Charles River Laboratories, Sulzfeld, Germany) at the age of 11–15 weeks with a mean weight 3.22 ± .27 kg were used for this study. All rabbits were housed in a pathogen-free animal facility, in rooms with laminar flow, constant temperature and constant humidity. Food and water were provided *ad libitum*. However, the rabbits were fasted 2 h before the measurements and blood glucose (BG) level, as well as animal weight was measured prior to MRI scans (Table 1). Imaging was performed at the same daytime under deep-sedation of rabbits by subcutaneous injections of medetomidin hydrochlorid (Cepetor, 200 mg/kg body weight) and Ketamin (Anesketin, 300 mg/kg body weight). Immediately after experimental imaging, euthanasia was performed by intravenous injection of pentobarbital sodium (Narcoren, 300 mg/kg body weight) and liver explantation was conducted. Tissues were dissected and snap frozen in liquid nitrogen for further proteomics analysis.

**TABLE 1:** Descriptive data of rabbits. Parameters with group mean values given as mean ± standard deviation for all 19 rabbits, including weight, BG, SWS, PR, SUV. BG, blood glucose; SWS, shear wave speed; PR, penetration rate; SUV, standardized uptake value.

ID	Weight in kg	BG in mg/dl	SWS in m/s	PR in m/s	SUV
3	3.6	107	1.45 ± .11	1.04 ± .33	2.50 ± .21
1	2.6	178	1.65 ± .16	1.16 ± .26	2.26 ± .36
15	3.5	168	1.60 ± .15	.99 ± .31	3.06 ± .28
4	3.0	139	1.51 ± .14	.96 ± .23	2.34 ± .37
9	3.5	154	1.70 ± .17	1.01 ± .35	3.00 ± .36
16	3.3	187	1.56 ± .13	.87 ± .24	2.88 ± .21
4	3.4	140	1.39 ± .10	.84 ± .22	2.73 ± .18
5	3.4	158	1.41 ± .13	.88 ± .28	2.74 ± .16
2	3.1	184	1.70 ± .12	1.21 ± .39	2.82 ± .10
22	3.2	132	1.65 ± .14	1.06 ± .29	2.89 ± .18
13	3.6	191	1.64 ± .16	1.03 ± .28	
7	3.2	150	1.69 ± .12	1.03 ± .30	
6	3.5	135	1.57 ± .15	1.09 ± .44	
14	2.9	159	1.56 ± .11	1.03 ± .38	
12	3.1	123	1.64 ± .12	1.02 ± .29	
8	2.9	144	1.55 ± .16	.83 ± .29	
18	3.2		1.68 ± .11	.99 ± .35	
17	3.2		1.71 ± .16	1.00 ± .44	
19	3.2		1.51 ± .14	.80 ± .18	
Mean	3.23 ± .26	153.06 ± 24.1	1.59 ± .1	.99 ± .11	2.72 ± .27

### 2.2 Imaging

#### 2.2.1 MRI

All experiments were performed on a clinical 3-Tesla hybrid PET/MR scanner (Magnetom Biograph mMR, Siemens Healthineers, Erlangen, Germany) with a 20-channel head coil. Rabbits were positioned head first in prone position. For anatomic orientation, a transversal T1-weighted fat-saturated Dixon sequence (repetition time [TR] 4.76, echo time [TE] 1.49 ms, matrix 512 × 512 mm, voxel size .5 × .5 × 2.0 mm³ and coverage of the complete liver) was performed in the abdominal region of the rabbits before MRE and PET scans.

#### 2.2.2 Multifrequency MRE

**FIGURE 1:**
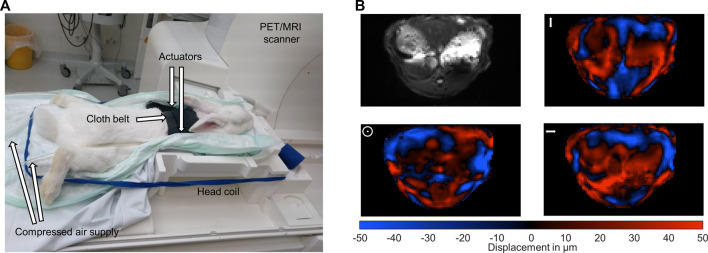
Experimental setup. **(A)** Multifrequency MRE setup for abdominal investigations in rabbits in a 3-Tesla PET/MRI scanner using a 20-channel head coil. Harmonic oscillations are generated by pressurized air pulses transmitted *via* tubes into two plastic actuators. The two actuators are placed in the upper abdominal region of the rabbits. **(B)** Representative MRE magnitude image (greyscale) and wave fields (color scale) for the three motion encoding components at 60 Hz vibration frequency. Mean displacement for the three motion encoding components was 31 µm. The symbols ↕, ʘ, ↔ illustrate head-feet, ventral-dorsal and left-right, deflections, respectively.

The used multifrequency MRE setup was similar to previous *in vivo* studies in patients with driver fixation adapted to rabbits (Shahryari et al., 2019; Shahryari et al., 2021). 8 phase offsets equally distributed over full vibration cycle of 40 Hz, 50 Hz, 60 Hz, 70 Hz and 80 Hz mechanical vibrations were recorded for all three Cartesian-motion field directions in a transversal plane using a single-shot spin-echo echo-planar-imaging (EPI) sequence (Tzschätzsch et al., 2016; Dittmann et al., 2017). Oscillations were induced by two pressurized air drivers attached onto the upper abdominal region of the rabbits. The drivers were powered by air pulses of .2 bar maximum amplitude. A cloth belt around the abdomen was used to improve the contact of the drivers to the rabbits and the efficiency of shear wave excitation. Vibrations were started 2 s prior to image acquisition to ensure a steady-state flux of harmonic shear wave energy. Imaging parameters were: TE 40 ms, TR 1000 ms, matrix 104 × 60, voxel size 1.55 × 1.55 × 5 mm³, 6 contiguous slices in a transversal view, 4 averages, parallel imaging with GRAPPA-factor 2, motion encoding gradient (MEG) frequency 78.61 Hz for all vibration frequencies, MEG amplitude 42 mT/m with first order moment nulling. Total MRE acquisition time was approximately 8 min. Cloth belt and actuators were removed prior to PET image acquisition. Figures 1A, B show the experimental MRE setup along with wave field components at 60 Hz vibration frequency.

#### 2.2.3 PET

PET measurement was conducted in a subgroup of 10 rabbits injecting ^18^F-FDG as radiotracer intravenously (mean 75.88 ± 14.35 MBq, min 38.59 MBq, max 91.42 MBq). The PET scan was performed, covering the entire thorax and abdomen. PET image reconstruction was conducted by ordered-subset expectation maximization (OSEM) algorithm with 3 iterations and 21 subsets, 512 × 512 × 127 image matrix and 1 × 1 × 2 mm³ voxel size. Ultrashort echo-time sequence (UTE) implemented by the vendor was used for attenuation and scatter correction (AC).

#### 2.2.4 Image processing

MRE data were processed in MATLAB Release 2021a (The Mathworks Inc. Natick, MN, United States) using wavenumber-based inversion method (k-MDEV) (Tzschätzsch et al., 2016) publicly available at https://bioqic-apps.charite.de (Meyer et al., 2022b). A Butterworth low-pass filter of order 3 with a threshold of 250 m^−1^ was used to suppress noise prior to image unwrapping (Herthum et al., 2022) while a spatial bandpass Butterworth filter of order 3 with thresholds of 15 m^−1^ and 300 m^−1^ was used for directional filtering (Herthum et al., 2022). Frequency-compound maps of shear wave speed (SWS in m/s) and penetration rate (PR in m/s) were computed as surrogates for tissue stiffness and inverse viscosity (Reiter et al., 2020). MRE magnitude images, SWS-, PR- and attenuation-corrected PET maps were further analyzed and used for manually drawing 3D regions of interest (ROIs) covering the liver. Mean liver SWS and PR values were calculated for each rabbit. Mean standardized uptake values (SUV) was calculated for 10 rabbits based on activity concentration (A(t)) in kBq/mL, body weight (W) in kg, injected dose (ID) in MBq, dose and decay correction factor d derived by the half-life of the radiotracer ^18^F-FDG and the time delay between injection and start of the measurement:

$${SUV}_{VOI}=\frac{A\left(t\right)∙W}{ID∙d}$$

### 2.3 Shotgun proteome profiling and data analysis

#### 2.3.1 Proteomics sample preparation with label-free quantification (LFQ)

Between 11 and 100 mg of each liver tissue were homogenized under denaturing conditions with a FastPrep (two times for 60 s, 6.5 m × s^−1^) in 1 mL of a fresh buffer containing 3 M guanidinium chloride (GdmCl), 10 mM Tris (2-carboxyethyl)phosphine, 20 mM chloroacetamide and 100 mM Tris-HCl pH 8.5. Lysates were boiled at 95°C for 10 min in a thermal shaker, followed by sonication for 10 min and centrifuged at 10,000 rcf for 5 min at 4°C. The supernatant was transferred into new protein low binding tubes (Eppendorf, Germany). 30°µg protein per sample were diluted to 1 M GdmCl by adding 10% acetonitrile and 25 mM Tris, 8.5°pH, followed by a Lys C digestion (Roche, Basel, Switzerland; enzyme to protein ratio 1:50, MS-grade) at 37°C for 2 h. This was followed by another dilution to .5 M GdmCl and a tryptic digestion (Roche, 1:50) at 37°C, at 800 rpm, and overnight. Subsequently, peptides were desalted with C18 columns and reconstituted in 2% formic acid in water and further separated into five fractions by strong cation exchange chromatography (SCX, 3 M Purification, Meriden, CT). Eluates were first dried in a SpeedVac, then dissolved in 5% acetonitrile and 2% formic acid in water, briefly vortexed, and sonicated in a water bath for 30 s prior to injection to nano-Liquid chromatography–mass spectrometry (LC-MS).

#### 2.3.2 LC-MS/MS instrument settings for shotgun proteome profiling and data analysis

LC-MS/MS was carried out by nanoflow reverse-phase liquid chromatography (Dionex Ultimate 3000, Thermo Scientific) coupled online to a Q-Exactive HF Orbitrap mass spectrometer (Thermo Scientific), as reported previously (Gielisch and Meierhofer, 2015). Briefly, the LC separation was performed using a PicoFrit analytical column (75 μm ID × 50 cm long, 15 µm Tip ID; New Objectives, Woburn, MA) in-house packed with 3 µm C18 resin (Reprosil-AQ Pur, Dr. Maisch, Ammerbuch, Germany). Peptides were eluted using a gradient from 3.8% to 38% solvent B in solvent A over 120 min at a 266 nL per minute flow rate. Solvent A was .1% formic acid and solvent B was 79.9% acetonitrile, 20% H_2_O, and .1% formic acid. For the IP samples, a 1-h gradient was used. Nanoelectrospray was generated by applying 3.5 kV. A cycle of one full Fourier transformation scan mass spectrum (300–1750 m/z, resolution of 60,000 at m/z 200, automatic gain control (AGC) target 1 × 10^6^) was followed by 12 data-dependent MS/MS scans (resolution of 30,000, AGC target 5 × 10^5^) with a normalized collision energy of 25 eV.

Raw MS data were processed with MaxQuant software (v 1.6.1.43) and searched against the *Oryctolagus cuniculus* (rabbit) proteome database UniProtKB (UP000001811) with 21,178 entries, released in September 2019. Parameters of MaxQuant database searching were a false discovery rate (FDR) of .01 for proteins and peptides, cysteine carbamidomethylation was set as fixed modification, while N-terminal acetylation and methionine oxidation were set as variable modifications. The mass spectrometry proteomics data have been deposited to the ProteomeXchange Consortium *via* the PRIDE (Perez-Riverol et al., 2022) partner repository with the dataset identifier PXD036659.

### 2.4 Assessment of metabolic capacities

**FIGURE 2:**
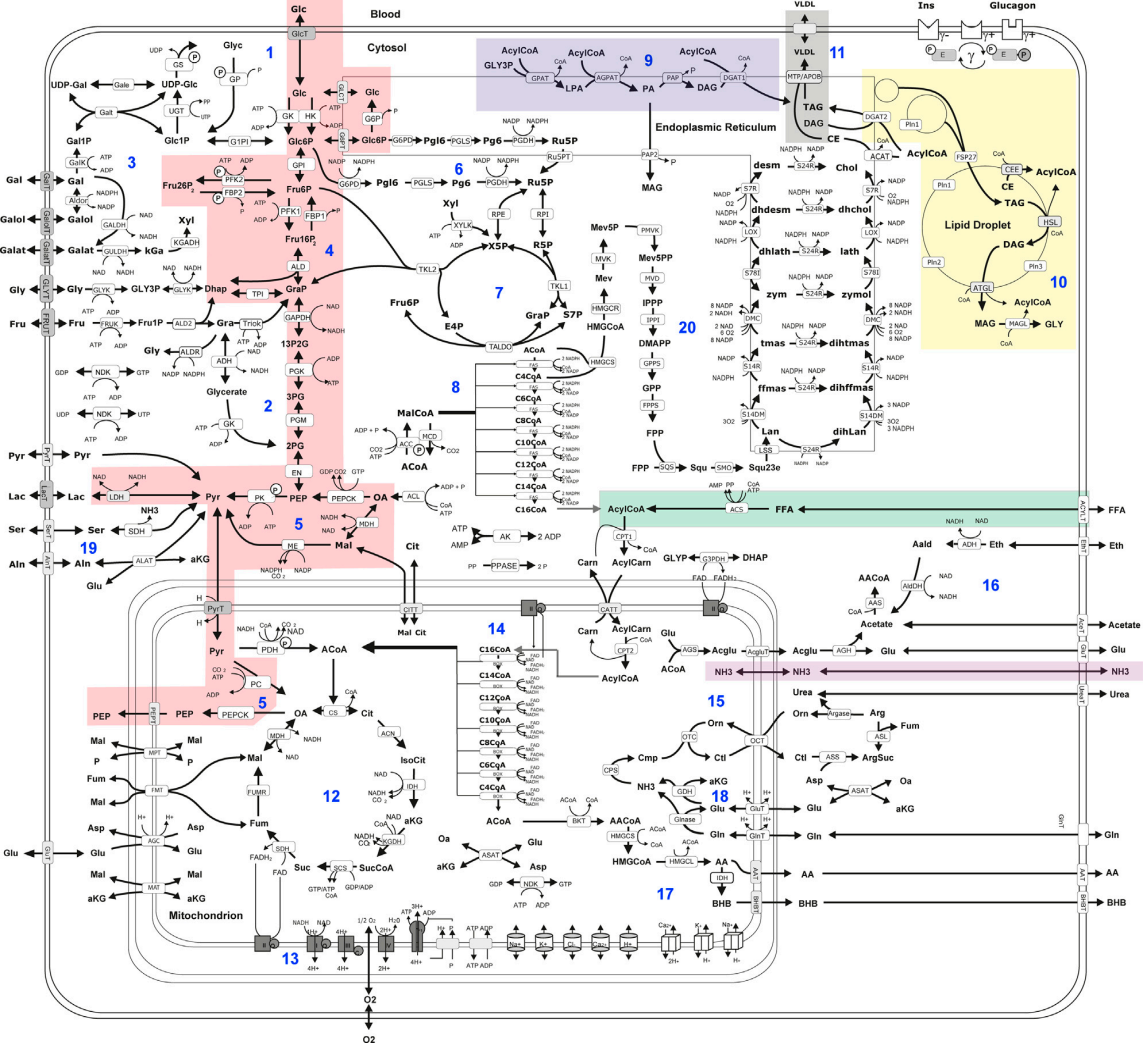
Scheme of liver metabolism model adapted from Bernd et al. (Berndt et al., 2018a). 16. Reactions and transport processes between compartments are symbolized by arrows. Single pathways are numbered in blue: (Mathew et al., 2022) glycogen metabolism (Bussler et al., 2017), fructose metabolism (Lonardo et al., 2015), galactose metabolism (Sivell, 2019), glycolysis (Wong and Singal, 2020), gluconeogenesis (Caldwell and Argo, 2010), oxidative pentose phosphate pathway (Seyama and Kokudo, 2009), non-oxidative pentose phosphate pathway (Lalazar et al., 2008), fatty acid synthesis (Vos et al., 2014), triglyceride synthesis (De Gasperi et al., 2016), synthesis and degradation of lipid droplets (Gorowska-Kowolik et al., 2017), synthesis of very low density lipoprotein (vldl) (Berndt et al., 2021a), tricarbonic acid cycle (Berndt et al., 2021b), respiratory chain and oxidative phosphorylation (Berndt et al., 2020), β-oxidation of fatty acids (Berndt et al., 2019), urea cycle (Berndt et al., 2018a), ethanol metabolism (Kespohl et al., 2020), ketone body synthesis (Boyd et al., 2020), glutamate and glutamine metabolism (Ratziu et al., 2005); serine and alanine utilization (Chalasani et al., 2018), cholesterol synthesis. Lipid droplet synthesis and degradation pathway include *de novo* synthesis of lipid droplets, lipid droplet filling, lipid droplet growth and fusion as well as lipid droplet degradation in dependence of regulatory surface proteins 45. Small cylinders and cubes symbolize ion channels and ion transporters. Double arrows indicate reversible reactions, which may proceed in both directions according to the value of the thermodynamic equilibrium constant and cellular concentrations of their reactants. Reactions are labeled by the short names of the catalyzing enzyme or membrane transporter given in the small boxes attached to the reactions arrow. Highlighted metabolic pathways show association with MRE imaging parameters: gluconeogenesis (red); triacylglycerol (tag) synthesis (blue); tag storage (yellow); fatty acid uptake (green); very low density lipoprotein (vldl) export (grey); ammonia uptake (purple). Metabolites are denoted by their short names. Full names of metabolites and kinetic rate laws of reaction rates are outlined in Berndt et al. (2018a) and Wallstab et al. (2017).

Hepatic metabolic capacities were assessed using HEPATOKIN1 (Berndt et al., 2018a) in combination with a molecular-resolution model of hepatic lipid droplet metabolism (Wallstab et al., 2017). It comprises the major metabolic pathways of carbohydrate, lipid and amino-acid metabolism in hepatocytes (see Figure 2). Electrophysiological processes at the inner mitochondrial membrane describing the generation and utilization of the proton motive force were modelled by kinetic equations of the Goldman-Hodgkin-Katz type (Berndt et al., 2015). Hormone-dependent regulation of the liver metabolism by reversible enzyme phosphorylation was taken into account by a phenomenological transfer function (Bulik et al., 2016). Individual model instantiations were generated based on proteomic profiles as described in Berndt et al. (2019).

Physiological metabolic functions were defined as maximal fluxes obtained under a wide range of physiological conditions (3–12 mM plasma glucose), where plasma metabolite concentrations are not independent from each other. The interdependence between plasma glucose, plasma hormone and plasma fatty acid concentration was taken into account by using experimentally determined transfer functions (Bulik et al., 2016; Berndt et al., 2018a).

### 2.5 Statistical analysis

Statistical analysis and cluster analysis were done MATLAB Release 2021a (The MathWorks, Inc., Natick, MA, United States) with the bioinformatics toolbox. Group values were checked for normality based on quantile-quantile plots (qqPlots) with a 95% confidence interval margins. For normal distributed grouped values, unpaired two-sided students’ *t*-test was used to calculate group differences. Otherwise, Wilcoxon signed-ranked test was used. Statistical analysis was performed using R version 4.0.2 (R-Foundation, Vienna, Austria). The level of significance was *p* < .05. Cluster analysis was performed using clustergram function. Row-wise normalization was used transforming values so that the mean of each metabolic function is 0 and the standard deviation is 1.

## 3 Results

### 3.1 Metabolic modelling

For the assessment of metabolic capabilities, we used personalized kinetic models to compute steady-state load characteristics describing the metabolic functions under a wide range of physiological conditions ranging from fasted state (low glucose, high fatty acids, low insulin and high glucagon) to a fed state (high glucose, low fatty acids, high insulin, low glucagon). The remaining plasma nutrients (e.g., lactate, amino acids, ketone bodies, ammonia) were kept fixed at mean diurnal values (see Section 2.4 for more details).

**FIGURE 3:**
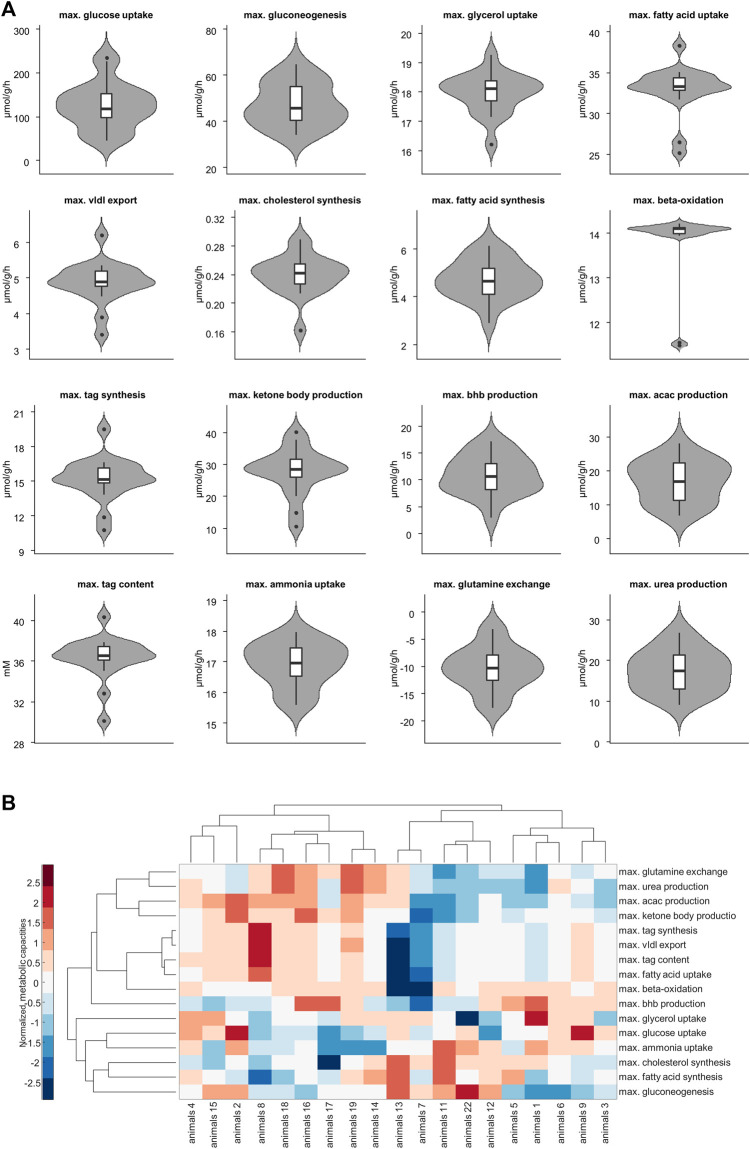
**(A)** Selected metabolic capacities. Each plot consists of 19 samples. **(B)** Hierarchical cluster analysis of the assessed 16 metabolic capacities. Values are normalized so that for each function the mean is 0 and the standard deviation is 1. Red and blue colors indicate increased and decreased metabolic function for each sample compared to the overall mean. acac, acetylacetone; tag, triacylglycerol; vldl, very low density lipoprotein, bhb; β-hydroxybutyrate.

Figure 3A shows the panel of investigated metabolic capacities. Our analysis revealed strong metabolic differences among the individual livers. Individual livers differed in metabolic functions regarding carbohydrate metabolism, including glycolysis and gluconeogenesis, ketone body synthesis and fatty acid metabolism such as fatty acid uptake, lipoproteins production, triacylglycerol (tag) storage and fatty acid synthesis. We performed an unbiased cluster analysis to group the livers according to similarities in their metabolic functions. The cluster analysis depicted in Figure 3B shows that two main groups could be identified. Compared with the mean of all livers, metabolic cluster 2 is characterized by a less active metabolism of fatty acids, including reduced fatty acid uptake, reduced tag synthesis and storage as well as reduced ketone body synthesis. Instead, it has a higher synthetic capacity for glucose, fatty acids and cholesterol. Complementary, the metabolic cluster 1 has a more pronounced fatty acid metabolism and reduced biosynthetic activity than the mean of all livers.

**FIGURE 4:**
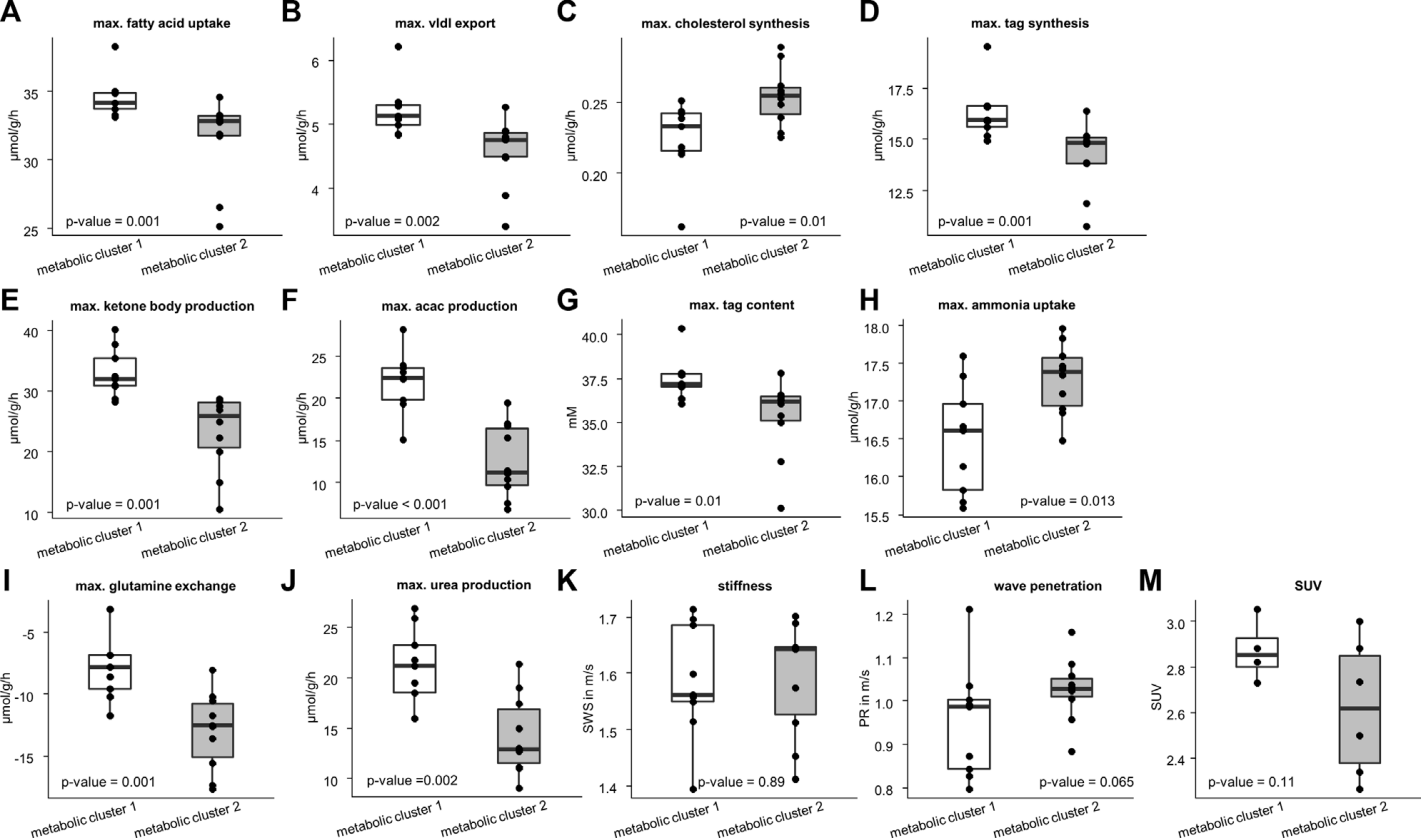
Differences in metabolic capability and imaging parameters between the two metabolic clusters. Cluster 1 consists of 10 samples and cluster 2 of 9 samples. Metabolic characteristics: **(A)** fatty acid uptake; **(B)** very low density lipoprotein (vldl) export; **(C)** cholesterol synthesis; **(D)** triacylglycerol (tag) synthesis; **(E)** ketone body production; **(F)** acetylacetone (acac) production; **(G)** max tag content; **(H)** ammonia uptake; **(I)** glutamine exchange; **(J)** max urea production. Imaging parameters: **(K)** shear wave speed (SWS); **(L)** wave penetration (PR); **(M)** standardized uptake value (SUV). Corresponding *p*-values based on two-sided *t*-test or Wilcoxon signed-rank test are given. The center line represents the median, the box represents the interquartile range, and the whiskers are defined by values within 1.5 times the interquartile range. Outliers are represented as dots.

We used the two metabolic clusters to define subgroups and investigated whether metabolic functions differ significantly between the two clusters. Cluster 1 consisted of 9 samples, whereas cluster 2 consisted of 10 samples. Figure 4 shows that the two groups differed significantly with respect to fatty acid uptake (34.7 ± 1.54 vs. 31.51 ± 3.1 μmol/g/h, *p* = .001), very low density lipoprotein (vldl) export (5.22 ± 0.42 vs. 4.56 ± .54 μmol/g/h, *p* = .002), cholesterol synthesis (.22 ± 0.03 vs. .25 ± .02 μmol/g/h, *p* = .01), tag synthesis (16.32 ± 1.35 vs. 14.15 ± 1.67 μmol/g/h, *p* = .001), ketone body production (32.91 ± 4.04 vs. 23.23 ± 6.31 μmol/g/h, *p* = .001), acetylacetone (acac) production (21.95 ± 3.6 vs. 12.53 ± 4.28, *p* < .001 μmol/g/h), tag content (37.48 ± 1.24 vs. 35.31 ± 2.24 mM, *p* = .01), ammonia uptake (16.49 ± .73 vs. 17.3 ± .46 μmol/g/h, *p* = .013), glutamine exchange (−7.54 ± 2.94 vs. −12.98 ± 3.12 μmol/g/h, *p* = .001), and urea production (20.96 ± 3.93 vs. 14.26 ± 3.88 μmol/g/h, *p* = .002).

**FIGURE 5:**
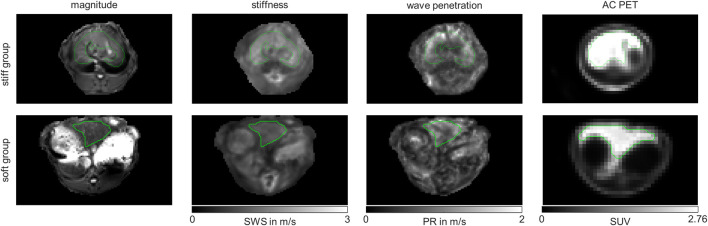
Representative MRE magnitude, stiffness indicated by shear wave speed (SWS), wave penetration indicated penetration rate (PR) and attenuation corrected (AC) positron emission tomography (PET) maps of *in vivo* rabbit liver. Shown are representative examples from the stiff liver (SWS>1.6 m/s) and soft liver (SWS<1.6 m/s) group. Maps are shown in grey-scale. Liver regions of interests (ROIs) are demarcated by green lines.

We also checked whether the metabolic subgroups are characterized by significant differences in imaging markers. Figure 5 shows a representative slice of MRE and PET imaging parameters of rabbit’s liver. SWS, PR and SUV values are given in Table 1. While no significant differences could be detected, PR shows a tendency to be lower in the livers with increased fatty acid metabolism and decreased biosynthetic capacities, hinting at a connection between non-invasive imaging markers and metabolic functionality.

Another approach to investigate the association of liver metabolism to liver mechanics was the clustering based on liver mechanics. Therefore, we divided the livers into two groups according to liver stiffness. Stiff livers were defined by SWS >1.6 m/s (*n* = 10), while soft livers were defined by a SWS <1.6 m/s (*n* = 9), corresponding to the median of all SWS values of the rabbits. Using this classification, we again compared metabolic functions and non-invasive imaging markers between these two groups to see, whether liver stiffness can be used to differentiate hepatic metabolic functionality.

**FIGURE 6:**
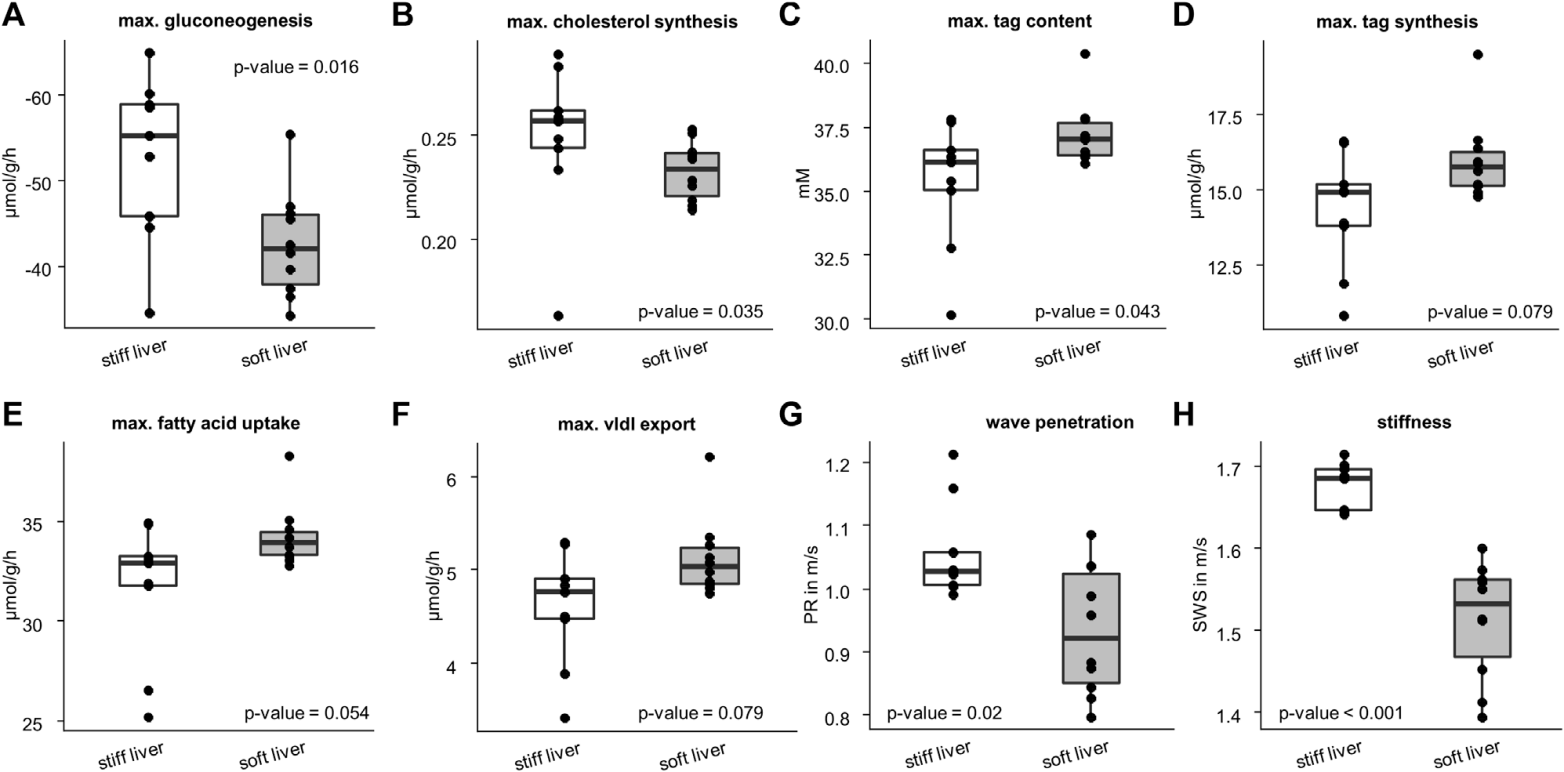
Differences in metabolic capacities and imaging parameters between the soft and stiff livers. Stiff group consist of 10 samples and soft group of 9 samples. Metabolic characteristics: **(A)** gluconeogenesis; **(B)** cholesterol synthesis; **(C)** triacylglycerol (tag) content; **(D)** tag synthesis; **(E)** fatty acid uptake; **(F)** very low density lipoprotein (vldl) export. Imaging parameters: **(G)** wave penetration (PR); **(H)** shear wave speed (SWS). Corresponding *p*-values based on two-sided *t*-test or Wilcoxon signed-rank test are given. The center line represents the median, the box represents the interquartile range, and the whiskers are defined by values within 1.5 times the interquartile range. Outliers are represented as dots.

**FIGURE 7:**
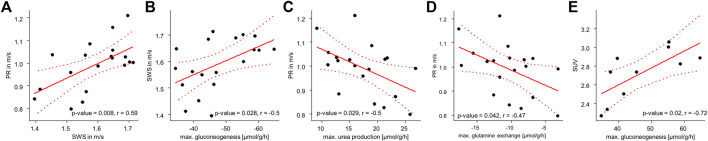
Correlation analyses of metabolic capabilities and MRE: **(A)** Correlation between SWS and PR; **(B)** SWS and gluconeogenesis; **(C)** PR and urea production; **(D)** PR and glutamine exchange and **(E)** SUV and gluconeogenesis. *p*- and *r*-values according to the linear regression model with *n* = 19 given in each panel. Red dotted lines indicate 95% confidence intervals of the linear model.

Figure 6 shows that stiff livers had a significantly higher capacity for gluconeogenesis (−52.81 ± 9.48 vs. −42.55 ± 6.17 μmol/g/h, *p* = .016), cholesterol synthesis (.25 ± .04 vs. .23 ± .01 μmol/g/h, *p* = .035), and tag content (35.32 ± 2.45 vs. 37.26 ± 1.25 mM, *p* = .043). Besides increased SWS, the parameter PR was also significantly higher in stiff livers than in soft livers (1.06 ± .08 vs. .93 ± .1 m/s, *p* = .02) and a linear correlation between SWS and PR was observed (*r* = .59, *p* = .008, Figure 7A).

### 3.2 Correlations of metabolic functions with MRE parameters

So far, we have shown, that metabolic functionality can be used to define metabolic subclasses in healthy livers and that these subclasses can be detected by MRE based on liver viscosity. Vice versa we showed, that differentiating between stiff and soft livers allows classification of metabolic functionality. While subgroups enable the metabolic and biomechanical classification of livers, the question remains, whether individual metabolic functions can possibly be assessed by MRE. To answer this question, we correlated individual metabolic liver functions with liver SWS and PR. SWS was significantly correlated with gluconeogenesis (*r* = −.5, *p* = .028, Figure 7B), while PR was correlated with urea production (*r* = −.5, *p* = .029, Figure 7C) and glutamine exchange (*r* = −.47, *p* = .042, Figure 7D). Interestingly, SUV was correlated with gluconeogenesis (*r* = −.72, *p* = .02, Figure 7E), although not with glucose uptake.

## 4 Discussion

In this study, we demonstrated for the first time the feasibility of MRE in small animals using a clinical hybrid PET/MRI scanner. This PET/MRE system allowed us to generate maps related to stiffness and viscosity, as well as ^18^F-FDG uptake simultaneously; this allowed the first-ever investigation of the correlation between metabolism and viscoelasticity. Furthermore, we harvested liver tissue after euthanasia for modeling liver metabolism of key metabolic markers for each individual rabbit.

The first important finding of our study was a surprisingly large heterogeneity in metabolic capacities among the healthy rabbit livers. Although all the rabbits were genetically identical and received the same nutrition, there were clear differences in fatty-acid metabolism, ketone-body synthesis and urea metabolism. While this is the first time we studied rabbit liver, variability in metabolic capacities were also shown in healthy mouse liver as well as human pediatric patients with moderate liver fibrosis (Berndt et al., 2021b; Berndt et al., 2022).

Direct evaluation of metabolic fluxes *in vivo* remains an expensive experimental challenge. Estimation of *in vivo* fluxes by measuring time series of labelled nutrients (Hasenour et al., 2015; Hui et al., 2020; Rahim et al., 2021) is restricted to the analysis of very few pathways and gives the average flux rate during the time of measurement, but it is not suited for monitoring metabolic fluxes over a longer period of varying physical activity and plasma profiles of nutrients. Furthermore, metabolic flux rates depend not only on the availability of the substrate of interest but also on the overall plasma nutrient and hormone composition making it hard to assess all the information necessary to define the observed metabolic state.

In our approach we assessed metabolic capacities rather than actual fluxes. We evaluated 16 different metabolic capacities under a wide range of physiological conditions ranging from fasted state (low glucose, high fatty acids, low insulin, and high glucagon) to a fed state (high glucose, low fatty acids, high insulin, low glucagon). The remaining plasma nutrients (e.g., lactate, amino acids, ketone bodies, ammonia) were kept fixed at mean diurnal values (see Berndt et al., 2018a for more details).

In this study, we circumvented these problems by evaluation of metabolic capacities based on protein abundances under pre-described conditions rather than actual fluxes. This allowed us to robustly assess the metabolic differences which are not prone to short-term variations due to varying metabolic conditions because metabolic enzymes are stable for hours (see Berndt and Holzhutter, 2018 and references within). It is important to note that the metabolic capacities assessed in this way do not themselves form a consistent metabolic state, since conditions under which they are realized are not the same. For example, maximal gluconeogenesis is reached under low glucose conditions, while maximal glycolysis is reached under high glucose conditions.

The differences in metabolic capacities allowed a rough classification of the livers into two clusters. Cluster 2 was characterized by reduced fatty-acid metabolism and increased biosynthetic capacity, while cluster 1 displayed the opposite metabolic characteristics. While statistically not significant, probably owing to the low sample number, a similar trend was seen in ^18^F-FDG uptake as measured by PET (*p* = .11). Importantly, the two metabolic clusters showed a clear distinction in mechanical properties: cluster 1 was more viscous (lower PR) than cluster 2, albeit again without statistical significance (*p* = .065).

Alternatively, classification of livers according to their biomechanical properties may provide an indication of metabolic functions. Therefore, we classified livers according to their mechanical properties. The clustering in two groups based on the cutoff value of 1.6 m/s for the liver SWS agrees with SWS values of a healthy human liver, i.e., approximately 1.4 m/s for 30–60 Hz when considering the dispersion of SWS in the liver as analyzed by Morr et al. (2022). This biomechanical classification revealed that stiff livers differ significantly from soft livers in their metabolic capacities. Specifically, stiff livers have a markedly reduced tag content, while showing increased capacity for gluconeogenesis and cholesterol synthesis. This observation agrees with recent findings in adipose tissue, where a decrease in stiffness was associated with excessive accumulation of lipids (Abuhattum et al., 2022). In future studies, it would be interesting to investigate whether increased glucogenesis activity associated with increased liver stiffness is an early marker of pathologically altered metabolism toward NAFLD.

We also studied which liver functions are directly associated with mechanical parameters. Figure 7 shows that PR is significantly correlated with hepatic capacities of urea production and glutamine exchange. It is noteworthy that these metabolic capacities are not independent of one another, as ammonia detoxification can take place either by urea production or by glutamine synthesis.

One may speculate whether the observed correlations indicate a causal relationship between liver biomechanics and metabolic function. However, it is important to note that short-term physiological changes such as prandial state or perfusion rate induce acute changes in the liver’s mechanical properties within seconds or minutes (Yin et al., 2011; Jajamovich et al., 2014; Dittmann et al., 2017; Manduca et al., 2021; Obrzut et al., 2021). In contrast, alterations in metabolic capacities due to variable protein abundance represent long-term adaptations taking place over hours or days (Berndt and Holzhutter, 2018). To reduce the confounding influence of physiological fluctuations on the mechanical properties of the liver, rabbits were fasted before the measurements. Sedation of rabbits reduced heart rate and breathing frequency while physiological short-term effects should have averaged out during the 8 min MRE examination time. Overall, the coefficient of variation of liver stiffness was 6% which is lower than in other MRE studies in healthy rabbit livers (14%–42%, [Zou et al., 2016; Zou et al., 2020; Zou et al., 2022]), suggesting a minor effect of physiological fluctuations on the measured mechanical properties.

Long-term architectural changes related to metabolism are conceivable when one considers ultrastructural liver properties such as extracellular collagen deposition or cellular fat accumulation. As discussed earlier, these structural changes directly affect mechanical properties but could also be associated with metabolic capacities (Sasso et al., 2016; Hudert et al., 2019). For example, excessive storage of tag might indicate an increased capacity for fatty-acid uptake and concomitant production of lipoproteins from these fatty acids. Collagen deposition and liver fibrosis, on the other hand, might alter substrate availability by alterations in hepatic blood flow and perfusion pressure, and thereby alter the capacities of glucose metabolism that respond directly to glucose availability (Berndt et al., 2018b). Hence, a close interaction between liver structure and metabolism would result in a correlation between viscoelastic properties and clusters of metabolic markers, as indeed observed in this study.

It should be emphasized that our results were obtained from healthy rabbit livers. We anticipate larger effects in livers with diseases such as NAFLD, and would expect such diseases to be readily detectable by *in vivo* MRE. For example, PR was shown by MRE to correlate negatively with the degree of hepatic steatosis in children with NAFLD at the same time that liver stiffness increased (Hudert et al., 2019). This is consistent with our analysis, in which stiffer livers were associated with reduced fatty-acid metabolism and increased biosynthetic capacity. Hence, the results of our study may explain findings in the literature, and they put MRE forward as a potentially sensitive tool for further studies on the relationship between liver function and viscoelastic properties.

Although encouraging, our study has limitations. First, only a small portion of the liver was used for proteomic analysis, whereas the MRE investigation covered most of the liver. Regional heterogeneity or different fractions of portal and central liver regions could affect proteomics-based metabolic capacities (Berndt et al., 2021b). Because healthy livers were used, we assume no regional heterogeneity. Secondly, MRE measurements were performed under free breathing, which could have led to respiratory artifacts. However, the rabbits in our study were under deep sedation to minimize any respiration-induced distortions. Furthermore, in earlier work we showed that MRE performed under free breathing yields values similar to those obtained by MRE under breath hold (Shahryari et al., 2021). Thirdly, this pilot study included only 19 healthy rabbits. Future studies with more animals and disease models such as NASH are needed to further investigate the association of liver mechanical parameters with metabolism. Furthermore, PET measurements were performed only in a subset of rabbits for cost reasons. Therefore, metabolic cluster group 1 consists of only four data points, which does not allow statistical conclusions.

In summary, this study has revealed the relationship between liver metabolism analyzed by proteomics-based modeling and viscoelastic parameters measured with MRE *in vivo*. We demonstrate for the first time the feasibility of MRE in small animals using a clinical hybrid PET/MRI scanner. A surprisingly large heterogeneity of metabolic capacities was found in healthy rabbit livers, manifested by marked differences in carbohydrate metabolism, ketone body synthesis and fatty-acid metabolism. Biomechanical classification revealed that stiff livers are distinctly different from soft livers in that they have a lower capacity for triacylglycerol storage, while at the same time showing an increased capacity for gluconeogenesis and cholesterol synthesis. The sensitivity of MRE parameters to key metabolic functions in the liver suggests that MRE holds promise as a potentially useful noninvasive method for the assessment of liver function capacity in the future.

## Data Availability

The mass spectrometry proteomics dataset presented in this study can be found in an online repository. The name of the repository and accession number can be found below: https://www.ebi.ac.uk/pride/archive/, PXD036659. The raw imaging dataset presented in this study will be made available by the authors upon reasonable request.
